# Funding source and primary outcome changes in clinical trials registered on ClinicalTrials.gov are associated with the reporting of a statistically significant primary outcome: a cross-sectional study

**DOI:** 10.12688/f1000research.6312.2

**Published:** 2015-04-24

**Authors:** Sreeram V Ramagopalan, Andrew P. Skingsley, Lahiru Handunnetthi, Daniel Magnus, Michelle Klingel, Julia Pakpoor, Ben Goldacre

**Affiliations:** 1 SVR Consulting, London, UK; 2 Imperial College Medical School, London, UK; 3 Medical Research Council Functional Genomics Unit and Department of Physiology, Anatomy and Genetics, University of Oxford, Oxford, UK; 4 School of Social and Community Medicine, University of Bristol, Bristol, UK; 5 Department of Epidemiology, London School of Hygiene and Tropical Medicine, London, UK

**Keywords:** clinical trials, funding, primary outcome

## Abstract

**Background**: We and others have shown a significant proportion of interventional trials registered on ClinicalTrials.gov have their primary outcomes altered after the listed study start and completion dates. The objectives of this study were to investigate whether changes made to primary outcomes are associated with the likelihood of reporting a statistically significant primary outcome on ClinicalTrials.gov.

**Methods**: A cross-sectional analysis of all interventional clinical trials registered on ClinicalTrials.gov as of 20 November 2014 was performed. The main outcome was any change made to the initially listed primary outcome and the time of the change in relation to the trial start and end date.

**Findings**: 13,238 completed interventional trials were registered with ClinicalTrials.gov that also had study results posted on the website. 2555 (19.3%) had one or more statistically significant primary outcomes. Statistical analysis showed that registration year, funding source and primary outcome change after trial completion were associated with reporting a statistically significant primary outcome **.**

**Conclusions:** Funding source and primary outcome change after trial completion are associated with a statistically significant primary outcome report on clinicaltrials.gov.

## Introduction

Clinical trials provide the principal method with which to assess the effectiveness of therapeutic strategies ^1^. An important principle in the good conduct of clinical trials is that a summary of the trial protocol, with a pre-defined primary outcome, should be freely available before the study commences ^1^. In February 2000, the United States (US) Food and Drug Administration (FDA) created an online clinical trials registry named ClinicalTrials.gov ^2^. We and others have shown a significant proportion of interventional trials registered on ClinicalTrials.gov have their primary outcomes altered after the listed study start and completion dates ^3, 4^. In this extended analysis, we sought to investigate whether changes made to primary outcomes are associated with the likelihood of reporting a statistically significant primary outcome on ClinicalTrials.gov.

## Methods

We used R ( http://cran.r-project.org/web/packages/rclinicaltrials/vignettes/basics.html) to download data from all completed interventional clinical studies registered with ClinicalTrials.gov as of 20th November 2014, as previously described ^3^. New to this study, we also downloaded data concerning study results for these trials; specifically the ‘p value’ fields from the ‘study results’ tab for primary outcomes.

We searched for potential non-inferiority studies by text searching for the word inferiority in the title.

Changes in primary outcomes were defined as previously described ^3^. A study was classified as not having a primary outcome changed if the original primary outcome was listed as ‘same as current’. Probable funding source was derived using the algorithm previously described ^3^.

A trial having a statistically significant primary outcome was defined as a trial having a P value less than 0.05 in the p value field in the study results tab for any primary outcome.

We used logistic regression to calculate odds ratios (ORs) and 95% confidence intervals (95% CI) for comparisons between significant primary outcome and non-significant primary outcome groups, using registration date, primary outcome change after study completion and funding source as explanatory variables. P-values <0.05 were interpreted as significant. Statistical analyses were conducted using the STATA 12.0.0 software.

## Results

Dataset of funding source, primary outcome changes and statistical significance of clinical trials registered on ClinicalTrials.org All clinical studies classified as ‘interventional studies’ registered with ClinicalTrials.gov as of 20 ^th^ November 2014 are shown. Probable funding source was derived using the algorithm previously described ^3^. A statistically significant primary outcome was defined as a trial having a P value less than 0.05. 1=yes; 0=no; blank=no info in all columns except “studyphase” and “sponsortype (0=public; 1=industry; 2=mixed). pom: primary outcome measure; sig: significance.

As of 20 November 2014, 13,238 completed interventional trials were registered with ClinicalTrials.gov that also had study results posted on the website. The trials were registered between 1999 and 2014 and 2555 (19.3%) had one or more statistically significant primary outcomes. There were 3934 (29.7%) trials classed as non-industry funded, 1569 (11.9%) as mixed and 7735 (58.4%) as industry funded. 12632 (95.4%) trials had a change in the primary outcome reported at initial registration; 12243 (92.5%) of these occurred after the trial completion date.

Statistical analysis showed that registration year, funding source and primary outcome change after trial completion were associated with the reporting a statistically significant primary outcome ( Table 1). Mixed funding and increased year of registration (i.e. more recent calendar time) were associated with a decreased odds of reporting a statistically significant primary outcome. A primary outcome change and industry funding were associated with an increased odds of reporting a statistically significant primary outcome. We identified 123 trials that had inferiority in the title. Removing these studies from the analysis did not materially change the results.

When including study phase in the analysis (10633 studies with study phase data available), mixed funding and registration year became non-significant.

**Table 1.** Association of funding status and primary outcome change after trial completion with reporting a statistically significant primary outcome.

	Odds Ratio (95% confidence interval)	P value
**Public funding**	1	
**Mixed funding**	0.79 (0.67–0.94)	0.008
**Industry funding**	1.39 (1.25–1.54)	<0.001
		
**No primary outcome** **change**	1	
**Primary outcome change** **after completion date**	1.53 (1.12–2.10)	0.008
		
**Registration year (per** **additional year)**	0.97 (0.95–0.99)	0.006

## Conclusions

We found that the reporting of statistically significant outcomes on ClinicalTrials.gov was more likely for trials with primary outcomes that had been changed and also those funded by industry. Previous studies have documented these associations ^5, 6^, and we confirm these using ClinicalTrials.gov data. There are limitations to our analyses- we have not investigated in any detail the nature of the primary outcome change and the potential effect this would have on the statistical analysis/outcomes. As discussed previously ^3^, some primary outcome changes that we have identified may be typographical/semantic and may not reflect actual changes to the nature of the outcome. We also did not look specifically to see whether a changed primary outcome was the one with a statistically significant finding, just whether a statistically significant finding was found for any primary outcome for the study. The vast majority of studies with results reported on ClinicalTrials.gov had a primary outcome change. This suggests that these trials are ones where the registrations have more diligent data updating. Nevertheless, this should be seen in equal measure for trials with and without statistically significant primary outcomes. In summary, funding source and primary outcome changes are associated with the reporting of statistically significant primary outcomes on ClinicalTrials.gov.

## Data availability

F1000Research: Dataset 1. Dataset of funding source, primary outcome changes and statistical significance of clinical trials registered on ClinicalTrials.org, 10.5256/f1000research.6312.d45056 ^7^
