# Supplementary material for: Funding source and primary outcome changes in clinical trials registered on ClinicalTrials.gov are associated with the reporting of a statistically significant primary outcome: a cross-sectional study
Source: F1000Res. 2015 Apr 24;4:80. Originally published 2015 Mar 26. [Version 2] doi: 10.12688/f1000research.6312.2 (PMC4431380; doi:10.12688/f1000research.6312.2)
Supplement: Peer review history

# Peer review: Referee response for version 2

**DOI:** 10.5256/f1000research.6895.r8561

**Published:** 2015-05-05

**Authors:** Deborah Korenstein

**Affiliations:** 1 Division of General Medicine, Mount Sinai School of Medicine, New York, NY, USA

The authors have adequately addressed my concerns.

I have read this submission. I believe that I have an appropriate level of expertise to confirm that it is of an acceptable scientific standard.

# Peer review: Referee response for version 2

**DOI:** 10.5256/f1000research.6895.r8472

**Published:** 2015-04-27

**Authors:** Janet Wale

**Affiliations:** 1 Cochrane Collaboration Consumer Network, Brunswick, VIC, Australia

The authors have clarified a number of the issues raised.

I have read this submission. I believe that I have an appropriate level of expertise to confirm that it is of an acceptable scientific standard.

# Peer review: Referee response for version 1

**DOI:** 10.5256/f1000research.6769.r8116

**Published:** 2015-04-02

**Authors:** Janet Wale

**Affiliations:** 1 Cochrane Collaboration Consumer Network, Brunswick, VIC, Australia

This Research Note is an extension of the authors' 2014 article (reference 3). It has a very clearly defined question, whether changes made to the primary outcomes are associated with statistically significant primary outcomes. The present data therefore includes only completed interventional studies on clinicaltrials.gov.

The second paragraph of the Methods section refers to the 2014 article. This is unhelpful, particularly as it is not clear from the 2014 article how 'changes in primary outcomes' are defined. The final paragraph of the Results section states that registration year, funding source and primary outcome change after trial completion were associated with a significant primary outcome - yet these are in opposite directions; and registration year is complex (looking at the data and 2014 article). That is brevity has taken over from clarity.

Some of the limitations are included in the Conclusions: what exactly the changes were ('semantics' versus actual change; whether the changed outcome was the statistically significant outcome reported). The authors have not gone on to analyse their results by phase of trial; if the trials are randomised controlled trials, or otherwise. Another important question is how many of the completed trials have reported their results within a set timeframe (one year/two years), that is what about the trials that have not reported their results?

Has the number of industry funded trials increased over time compared with mixed and public funded trials? In plain language, what is the extent of the problem?

I have read this submission. I believe that I have an appropriate level of expertise to confirm that it is of an acceptable scientific standard, however I have significant reservations, as outlined above.

# Peer review: Referee response for version 1

**DOI:** 10.5256/f1000research.6769.r8112

**Published:** 2015-03-30

**Authors:** Deborah Korenstein

**Affiliations:** 1 Division of General Medicine, Mount Sinai School of Medicine, New York, NY, USA

Ramagopalan and colleagues have expanded on their previous work to assess the relationship between changes in the primary endpoint on clinicaltrials.gov and both funding source and a “positive” trial result. The authors found that changes to the primary listed endpoint were associated with both industry funding and with a positive outcome.

Their sample included completed interventional clinical trials listed on clinicaltrials.gov. They defined trials as having a positive result if they had a listed p-value <0.05. This may be problematic since it appears that their sample included non-inferiority trials (though it is not clear how many) and for these trials a non-significant p-value may indicate a “positive” (or at least non-inferior) result. Since there have been growing numbers of non-inferiority trials published in recent years, this may be a substantial issue. The authors may want to consider identifying non-inferiority trials and considering their results differently, or at least reporting the prevalence of non-inferiority trials if possible.

Aside from this methodologic weakness the other methods are rather straightforward and clear. However, the authors found that 95.4% of trials had changed the primary outcome at some point during the registration period. In contrast, in their previous work the same authors found that 32% of trials registered with clinicaltrials.gov had changed the primary endpoint. The reason for this dramatic difference is not clear, and the authors do acknowledge that the vast majority of studies changed their primary endpoint and that many of the changes may have been trivial. Further, in spite of this surprising finding the authors still found significant associations. However, the near-total prevalence of changes to the primary endpoint certainly suggests that changing a primary endpoint in the registry is highly routine and likely does not reflect fundamental change to the study. This weakens the relevance of the findings.

I have read this submission. I believe that I have an appropriate level of expertise to confirm that it is of an acceptable scientific standard, however I have significant reservations, as outlined above.
